# 2-(1*H*-Benzimidazol-2-yl)-4,5,6,7-tetrahydro-2*H*-indazol-3-ol, a Benzimidazole Derivative, Inhibits T Cell Proliferation Involving H+/K+-ATPase Inhibition

**DOI:** 10.3390/molecules191117173

**Published:** 2014-10-24

**Authors:** Jin Liu, Ning Huang, Ning Li, Si-Nian Liu, Min-Hui Li, Hua Li, Xing-Yan Luo, Yan-Tang Wang, Li-Mei Li, Qiang Zou, Yang Liu, Tai Yang

**Affiliations:** 1 Department of Immunology, Chengdu Medical College, Chengdu 610500, China; E-Mails: jinliu19871987@163.com (J.L.); huangning3168@163.com (N.H.); lining6931@126.com (N.L.); liusinian123@163.com (S.-N.L.); limy876@aliyun.com (M.-H.L.); luoxingyan2006@163.com (X.-Y.L.); yt-wang@hotmail.com (Y.-T.W.); limeiligy@gmail.com (L.-M.L.); qiangzou99@gmail.com (Q.Z.); 2 Department of Oncology, Chengdu Military General Hospital, Chengdu 610083, China; E-Mail: Huali99@gmail.com

**Keywords:** benzimidazole derivative, T cell proliferation, H+/K+-ATPases, immunomodulatory

## Abstract

In this study, a benzimidazole derivative named BMT-1 is revealed as a potential immunomodulatory agent. BMT-1 inhibits the activity of H+/K+-ATPases from anti-CD3/CD28 activated T cells. Furthermore, inhibition the H+/K+-ATPases by use of BMT-1 should lead to intracellular acidification, inhibiting T cell proliferation. To explore this possibility, the effect of BMT-1 on intracellular pH changes was examined by using BCECF as a pH-dependent fluorescent dye. Interestingly, increases in the pHi were observed in activated T cells, and T cells treated with BMT-1 showed a more acidic intracellular pH. Finally, BMT-1 targeted the H+/K+-ATPases and inhibited the proliferative response of anti-CD3/CD28-stimulated T cells. A cell cycle analysis indicated that BMT-1 arrested the cell cycle progression of activated T cells from the G1 to the S phase without affecting CD25 expression or interleukin-2 (IL-2) production; treating IL-2-dependent PBMCs with BMT-1 also led to the inhibition of cell proliferation. Taken together, these findings demonstrate that BMT-1 inhibits the proliferation of T cells by interfering with H+/K+-ATPases and down-regulating intracellular pHi. This molecule may be an interesting lead compound for the development of new immunomodulatory agents.

## 1. Introduction

Benzimidazoles are heterocyclic aromatic organic compounds. These bicyclic compounds consist of fused benzene and imidazole moieties. This particular structure can form hydrogen bonds with enzymes and receptors that exhibit varied bioactivities *in vivo*. Benzimidazole derivatives are found in numerous commercial drugs used in the clinical treatment of many diseases. For example, astemizole is a benzimidazole derivative used to treat allergic rhinitis [1]. Omeprazole is a substituted benzimidazole used to treat gastric acid-related diseases [2]. Telmisartan is a benzimidazole derivative used to treat hypertension [3]. Albendazole is a benzimidazole derivative used to treat worm infestations [4]. The immunosuppressive properties of benzimidazole-based compounds are also very valuable, as several benzimidazole derivatives exhibit primary immunosuppressive activities [5,6], therefore, periodic efforts have been made to screen these compounds for such potential activity.

**Figure 1:**
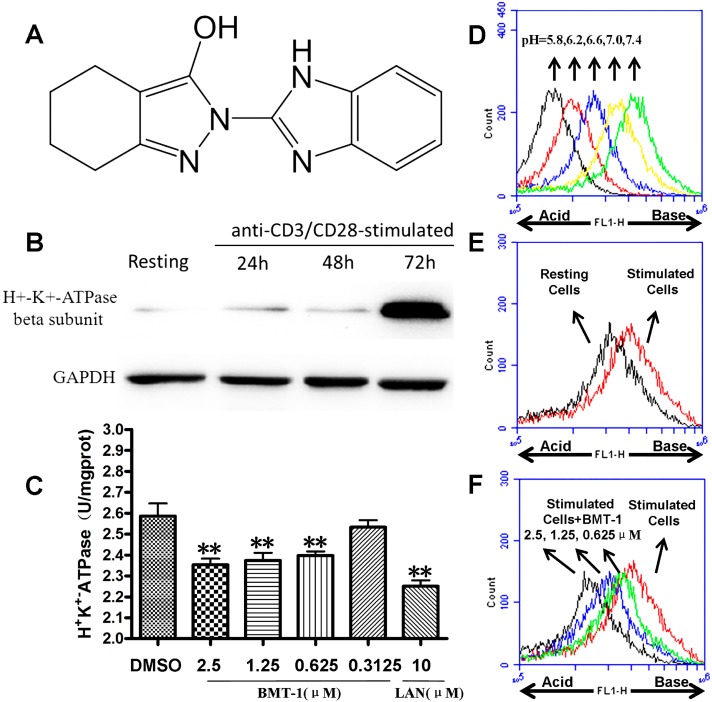
BMT-1 inhibits the activity of H+/K+-ATPases from anti-CD3/CD28 activated T cells. (**A**) Molecular structure of BMT-1. (**B**) Expression of H+-K+-ATPase in T cells identified by Western blot analysis. (**C**) BMT-1 inhibits the activity of H+/K+-ATPases in anti-CD3/CD28-stimulated T cells (n = 4). The concentration-response columns were analysed with GraphPad Prism software. Comparisons between groups and within groups were carried out using a one-way analysis of variance, and ** *p* < 0.01 was considered significant when compared to the control. (**D**) The pHi standard curve was developed using BCECF-AM as a pH-dependent fluorescent dye. (**E**) The increase in pHi was observed in anti-CD3/CD28 activated T cells. (**F**) Changes in the cytosolic pH induced by BMT-1 were evaluated in Anti-CD3/CD28-stimulated T cells treated for 6 h and loaded with the pH-sensitive fluorescent probe BCECF-AM. A more acidic intracellular pH was indicated by a lower FL1, and the intracellular pH drops in cells exposed to BMT-1. All experiments were performed twice in two independent experiments.

The H+/K+-ATPase or proton pump exchanges a proton with a potassium ion through a membrane. In the stomach, proton pump inhibitors (PPIs) block the H+/K+-ATPase in parietal cells that secrete acid into the gastric lumen. However, non-gastric cells also have proton pumps that might be inhibited by PPIs. For example, Ritter found immunoreactivity for a monoclonal antibody toward the H+/K+-ATPase in human neutrophils [7,8]. In our previous studies, we demonstrated the BMT-1 (Figure 1A) triggers apoptosis of multiple myeloma cells by H+/K+-ATPases inhibition [9]. Interesting, we found that H+/K+-ATPases have dramatically elevated expression in anti-CD3/CD28 stimulated T cells; if BMT-1, similar to other benzimidazole-based PPIs, inhibits T cell proton pumps, then BMT-1 treatment might interfere with T cell proliferation, facilitating the development and progression of inflammation. In this study, we demonstrate that BMT-1 inhibits the proliferative response of Anti-CD3/CD28-stimulated T cells associated the inhibition of proton pumps on T cells.

The stimulation of different cell types with growth factors is often accompanied by a rapid intracellular alkalinisation [10]. For example, multiple regression analyses of the data for both T and B lymphocytes indicated that the intracellular pH of cells in G0, G1, or G2 is around pH 7.2, while the intracellular pH of cells in the S phase of the cell cycle is around pH 7.4 [11]. In this study, an increase in pHi was observed in anti-CD3/CD28 activated T cells. However, little is known regarding the contribution of elevated intracellular pH on stimulated T and B lymphocytes. We proposed that the proliferation of T cells resulted in increased H+/K+-ATPase expression while relying on the efficient secretion of proton to avoid the intracellular accumulation of acids. We explored whether the BMT-1 inhibits H+/K+-ATPase activity to block proton traffic, resulting in proton accumulation and intracellular acidification that would terminate or limit the growth of T cells.

## 2. Results and Discussion

### 2.1. H+/K+-ATPases Inhibition Associated with the Acidification of Cytosolic pH in BMT-1 Treated Cells

The expression of H+/K+-ATPases in the T cells was identified using western blot analysis. In this study, we found that H+/K+-ATPases have dramatically elevated expression in anti-CD3/CD28 stimulated T cells over 72 h (Figure 1B).

To confirm whether the inhibition of H+/K+-ATPases activity was induced by BMT-1, the homogenised Anti-CD3/CD28-stimulated T cells (72 h) were treated with different concentrations of BMT-1 for 30 min, then the activities of H+/K+-ATPase and the OD value at 660 nm was detected according to the procedure of the H+/K+-ATPase kit.

Compared to the negative control group (DMSO), BMT-1 and the known inhibitor of H+/K+-ATPases (lansoprazole, LAN) inhibited H+/K+-ATPases from Anti-CD3/CD28-stimulated T cells (Figure 1C). The H+/K+-ATPases are ion pumps that use the energy from ATP hydrolysis to transport protons (H^+^) in exchange for potassium ions against their concentration gradients. Therefore, the inhibition of the H+/K+-ATPasess by BMT-1 may block H+ extrusion, resulting in proton accumulation and intracellular acidification; these effects terminate or limit the growth of the cancer cells [12,13]. To explore this possibility, the effect of BMT-1 on intracellular pH changes was examined by use of BCECF-AM as a pH-dependent fluorescent dye. As expected, the increase in pHi was observed in anti-CD3/CD28 activated T cells (Figure 1E), while the intracellular pH drops in cells exposed to BMT-1 (Figure 1F).

### 2.2. BMT-1 Inhibits T Cell Proliferation

**Figure 2:**
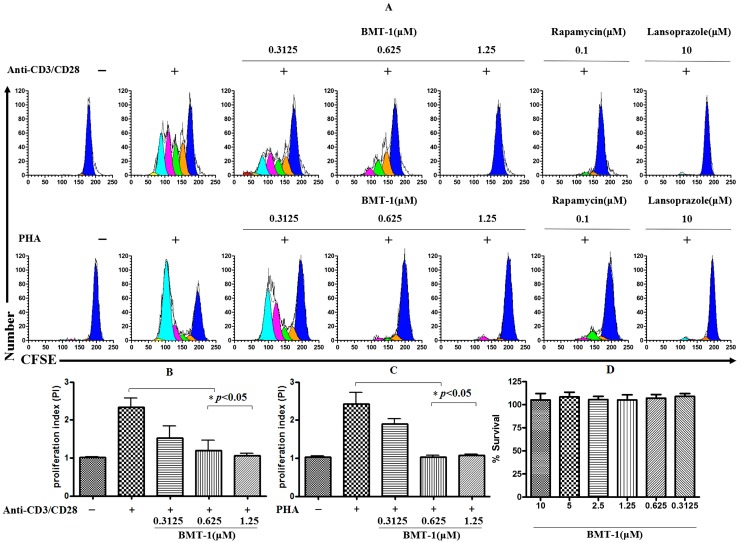
T cell proliferation was completely inhibited in the presence of BMT-1 (1.25 μM). (**A**) CFSE-labelled T cells were stimulated using PHA or anti-CD3/CD28 in the absence and presence of various concentrations of BMT-1. (**B**) The effect of BMT-1 on the proliferation index of CFSE-labelled anti-CD3/CD28 stimulated T cells. (**C**) The effect of BMT-1 on the proliferation index of CFSE-labelled PHA stimulated T cells. (**D**) Survival of PBMCs after 72 h of incubation with various concentrations of BMT-1. BMT-1 exhibits low toxicity toward lymphocytes. The results are presented as the means ± SD, n = 3, * *p* < 0.05 *vs.* control group (Anti-CD3-CD28/PHA induced and treated with vehicle).

To obtain information regarding the effect of BMT-1 on the proliferative capability of cells, purified human T cells were labelled with a fluorescein-based dye CFSE, and the cell proliferation was tracked via flow cytometry. This method is based on the sequential halving of cell fluorescence after each division, allowing the study of the division history of individual cells. As observed in Figure 2A, cells stimulated with anti-CD3/CD28 and PHA for 5 days showed 4 rounds of division. In contrast, cell proliferation was completely inhibited in the presence of BMT-1 (1.25 μM) and the known H+/K+-ATPase blocker Lansoprazole (10 μM). The proliferative index also was significant reduced (PI = 2.5 to 1) compared to T cells stimulated with anti-CD3/CD28 (Figure 2B) or PHA (Figure 2C). To examine the inhibition of anti-CD3/CD28 or PHA-stimulated T cells with BMT-1 that is not attributed to toxicity, we performed *in vitro* analyses using cultured PBMCs in the absence and presence of BMT-1. Figure 2D shows the survival rate of PBMCs cultured with various concentrations of BMT-1. No significant lymphotoxicity was observed after the exposure to BMT-1. These *in vitro* results confirm that BMT-1 exhibits low toxicity toward lymphocytes. Cumulatively, these results suggest that BMT-1 inhibits the growth of T cells stimulated by anti-CD3/CD28 or PHA using a mechanism unrelated to toxicity.

### 2.3. BMT-1 Decreases Anti-CD3/CD28 or PHA-Activated T Cell Proliferation by Arresting the Cell Cycle at the G0/G1 Phase

Because the proliferation of anti-CD3/CD28 or PHA-stimulated T cells was suppressed by BMT-1, we determined whether BMT-1 could block the anti-CD3/CD28 or PHA-induced cell cycle progression. Cells were activated with or without anti-CD3/CD28 or PHA in the presence or absence of BMT-1 for 3 days, and rapamycin was used as a positive control of T cell proliferation.

**Figure 3:**
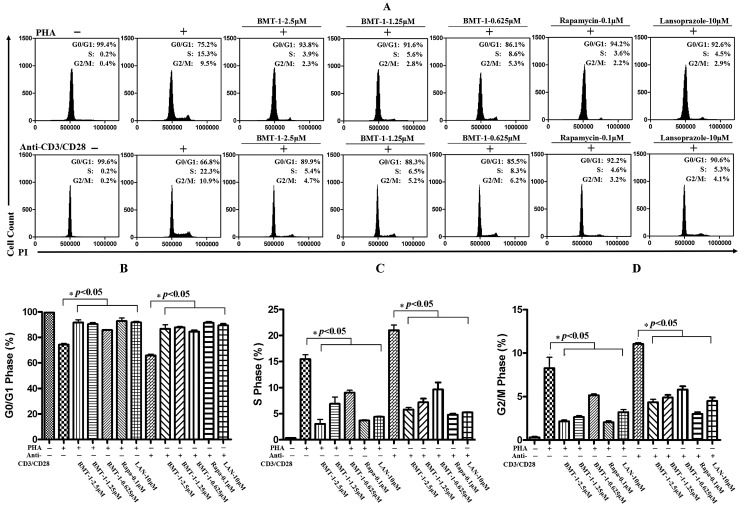
The stimulated cell cycle progression in T cells was largely blocked at the G0/G1 phase by BMT-1. (**A**) Representative flow cytometry analyses of cultured PHA or anti-CD3/CD28-stimulated T cell cycle in the presence of various concentrations of BMT-1. (**B**) The percentage of cells in the G0/G1 stage of the cell cycle. (**C**) The percentage of cells in the S stage of the cell cycle. (**D**) The percentage of cells in G2/M stage of the cell cycle. The data represent the means ± SD (n = 3); * *p* < 0.05 *versus* the control group (Anti-CD3-CD28/PHA induced and treated with vehicle) as determined by Student’s *t* test.

Afterwards, the cellular DNA was stained with propidium iodide and analysed with a flow cytometer. The results are shown in Figure 3. The fluorescence intensity of resting T cells was almost entirely in the G0/G1 phase. After the cells were stimulated with PHA or anti-CD3/CD28, their fluorescence intensity increased, and the cell distribution shifted from the G0/G1 phase to the S phase and G2/M phase. When PHA- or anti-CD3/CD28-activated T cells were treated with BMT-1 at 2.5 μM, the cell cycle progression was almost completely arrested at the G0/G1 stage. Therefore, the stimulated cell cycle progression in T cells was largely blocked at the G0/G1 phase by BMT-1.

### 2.4. BMT-1 has no Effect on IL-2 and CD25 Expression

**Figure 4:**
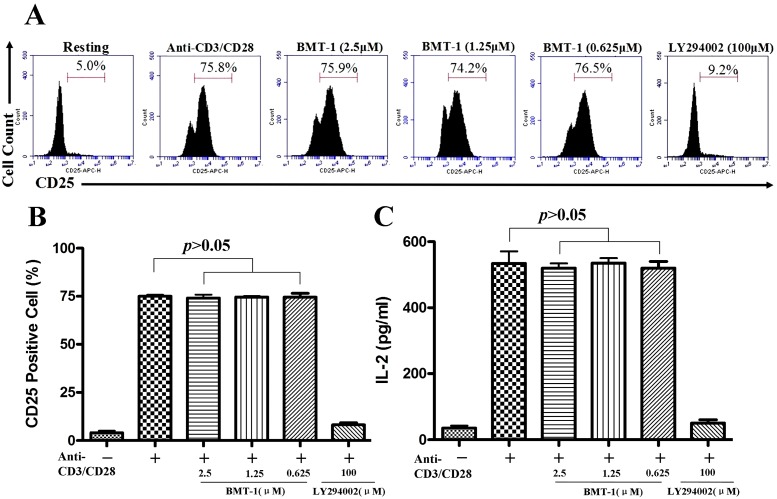
BMT-1 has no effect on CD25 and IL-2 expression. (**A**) The CD25 expression on lymphocytes was measured 48 h after human T cells were stimulated with anti-CD3/CD28 antibodies, and the data show that BMT-1 has no effect on the CD25 expression in T cells after TCR cross-linking. (**B**) The CD25 expression was evaluated using histogram analyses. The bars (mean ± SD of triple tests) represent the percentage of cells expressing CD25. There was no significant difference between the BMT-1 group and the stimulation group. As a positive control, LY294002 blocks the increase in CD25 expression in response to anti-CD3/CD28 costimulation. (**C**) BMT-1 has no effect on IL-2 generation. There was no significant difference between the BMT-1 group and the stimulation group.

After stimulation with anti-CD3 and anti-CD28 for 48 h, 75% of the T cells expressed the IL-2 receptor alpha chain (CD25). Because the cell cycle progression of T cells requires binding of IL-2 to the CD25 [14], and BMT-1 could block the anti-CD3/CD28 or PHA-induced cell cycle progression. Thus, it was important to determine whether CD25 and IL-2 expression was affected by BMT-1. In this result, LY294002, an inhibitor of Akt-1 phosphorylation could block the CD25 and IL-2 expression served as a positive control. BMT-1 showed no effect on the anti-CD3 and CD28 induced CD25 and IL-2 expression (Figure 4). As a positive control, LY294002 blocks the increase in CD25 and IL-2 expression in response to anti-CD3/CD28 costimulation. Taken together, these findings further demonstrate that BMT-1 inhibits the proliferation of T cells by interfering with H+/K+-ATPases and down-regulating intracellular pHi, and this inhibition is not due to the suppression of CD25 expression and IL-2 production.

### 2.5. BMT-1 Inhibits IL-2-Dependent PBMCs Proliferation

**Figure 5:**
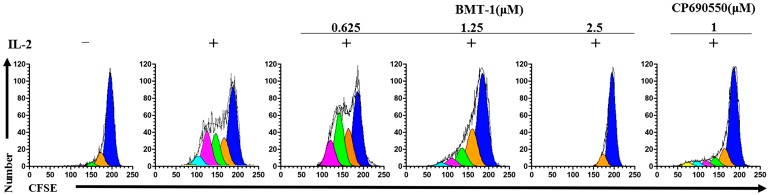
BMT-1 significantly suppressed IL-2-induced PBMCs proliferation. CFSE-labelled IL-2-dependent PBMCs were stimulated with IL-2 in the absence and presence of various concentrations of BMT-1.

Because BMT-1 did not affect IL-2 production or CD25 expression, we examined whether BMT-1 affected the IL-2 receptor-mediated proliferation of PBMCs. For this purpose, we assessed the proliferative response to IL-2-dependent PBMCs in the presence of BMT-1. In this experiment, CP-690550, an inhibitor of IL-2-stimulated phosphorylation of STAT5 could inhibit proliferation of IL-2-dependent PBMCs served as a positive control [15]. As shown in Figure 5, BMT-1 significantly inhibited proliferation of IL-2-dependent PBMCs in a dose-dependent manner. This inhibition was total at 2.5 μM (95% of inhibition), partial but still significant at 1.25 μM, and disappeared at 0.625 μM. IL-2 is a pro-inflammatory cytokine which promotes systemic inflammation [16,17]. BMT-1 also inhibits the IL-2-driven proliferation of activated T cells, and indicating that BMT-1 exhibits promising *in vitro* immunosuppressive activity.

### 2.6. Discussion

In this study, BMT-1 was observed to inhibit T cell proliferation. To understand the mechanisms of T cell proliferation induced by the small molecule BMT-1, the inhibitory effect of BMT-1 on H+/K+-ATPases was evaluated because BMT-1 is a benzimidazole derivative. It has already been observed that most well-known pharmaceuticals containing the benzimidazole core are H+/K+-ATPase pump or proton pump inhibitors (PPIs) [18]. By using a primary antibody against the H+/K+-ATPase beta-subunit (ATP4B), we were able to detect specific bands at ~70 kDa in both anti-CD3/CD28 activated and resting T cells (Figure 1B). This result indicated that BMT-1 may have intracellular compartments as their sites of action, thus causing H+/K+-ATPases inhibition. Thus, we investigated if BMT-1 possessed a characteristic of many other benzimidazole derivatives in being able to inhibit H+/K+-ATPase activity of T cells. The results showed that BMT-1 does indeed inhibit H+/K+-ATPases from anti-CD3/CD28 activated T cells (Figure 1C).

H+/K+-ATPases are ion pumps that use the energy of ATP hydrolysis to transport protons (H+) in exchange for potassium ions against their concentration gradients. When activated by stimuli such as anti-CD3/CD28 and PHA, the T cells undergo dramatic morphologic and metabolism changes. Therefore, we deduce that a major consequence of the altered T cell metabolism is that activated T-cells express more H+/K+-ATPase and rely on the efficient secretion of protons to avoid intracellular acid accumulation. Thus, the inhibition of H+/K+-ATPases by BMT-1 could block H+ extrusion, resulting in proton accumulation and intracellular acidification, which would terminate or limit the T-cell proliferation. As expected, the intracellular pH drops in cells exposed to BMT-1 (Figure 1F). This decreased pHi was caused by downstream of H+/K+-ATPases inhibition, similar to previous studies [19,20].

Changes in intracellular pH regulate many cell behaviours, including proliferation, migration, and transformation. Increases in pHi permit growth factor-induced cell proliferation [21,22]. However, our understanding of how physiological changes in pH affect T cell proliferation is limited [23]. Interesting, the increases in pHi were observed in anti-CD3/CD28 activated T cells in this study. Because cytosolic pH homeostasis is tightly regulated, dramatic differences in cell behaviour are driven by relatively small changes in pHi. Therefore, our results agree with previous reports showing that increased pHi values induce proliferation of immune and other cancer cells [10,11,22,24]. Compared to the increased pHi values, intracellular acidification could induce inhibition of proliferation for cells [25,26]. These activities result in an aberrant pH homeostasis critical for T cell proliferation and may be an important target for therapeutic purposes.

In this study, we found that BMT-1 significantly inhibited the proliferation of PHA-activated T cells in a dose-dependent manner. More importantly, the compound exhibited no cytotoxicity at 10 μM. This quality makes BMT-1 a potential candidate for drug development because low toxicity and high efficacy are the main criteria used to select lead compounds for drug discovery [27]. Furthermore, because the proliferation of anti-CD3/CD28-stimulated T cells was suppressed by BMT-1, we sought to determine whether BMT-1 could block the anti-CD3/CD28-induced cell cycle. Our studies showed that almost all of the non-stimulated T cells existed at the G0/G1 phase and that anti-CD3/CD28 induced entry into the cell cycle, while BMT-1 blocked anti-CD3/CD28-induced progression of the cell cycle from the G1 transition to the S phase.

IL-2 can up-regulate the CD25 expression at high concentrations [28], and the cell cycle progression of T cells requires binding of IL-2 to the CD25. Thus, it was important to determine whether IL-2 production was affected by BMT-1. Our results show that BMT-1 has no effect on IL-2 generation. Afterward, we examined whether BMT-1 affected IL-2 receptor-mediated PBMCs proliferation. For this study, we used IL-2-dependent PBMCs as a model system. Treating IL-2-dependent PBMCs with BMT-1 led to a dose-dependent inhibition of cell proliferation. As a pro-inflammatory cytokine, IL-2 is necessary for the growth, proliferation, and differentiation of T cells that become “effector” T cells. BMT-1 also inhibits the IL-2-driven proliferation of activated T cells, contributing to the development and progression of inflammation and indicating that BMT-1 exhibits promising *in vitro* immunosuppressive activity. This molecule may be an interesting lead compound for the development of new immunomodulatory agents.

## 3. Experimental Section

### 3.1. Chemicals

2-(1*H*-Benzimidazol-2-yl)-4,5,6,7-tetrahydro-2*H*-indazol-3-ol (BMT-1, purity>99%) was obtained from Chembridge, Inc. (San Diego, CA, USA). DMSO (Sigma-Aldrich Co., St. Louis, MO, USA) was used to dissolve BMT-1. Rapamycin was obtained from LC Laboratories (Woburn, MA, USA). The known H+/K+-ATPase blocker Lansoprazole was obtained from Sigma (Sigma-Aldrich Co.).

### 3.2. Antibodies and Reagents

Anti-human IL-2 purified, anti-human IL-2 biotin, anti-human CD3 affinity purified and anti-human CD28 affinity purified (costimulatory) antibodies were purchased from eBioscience (San Diego, CA, USA). phytohemagglutinin (PHA) was purchased from Sigma-Aldrich Co.

### 3.3. Cell Isolation and Culture

The blood donors were healthy individuals who provided informed consent. Peripheral blood mononuclear cells (PBMCs) were isolated via Lymphoprep density-gradient centrifugation (Nycomed AS, Oslo, Norway). The cells were resuspended in RPMI 1640 culture medium (Gibco, Gaithersburg, MD, USA) supplemented with 10% foetal bovine serum (Gibco) and 2 mM L-glutamine. The CD3+ T cells were negatively selected from PBMCs using immunomagnetic beads to deplete the non-T cells according to the manufacturer’s instructions (Miltenyi Biotec, Bergisch Gladbach, Germany). The purity of the resulting T cell populations was examined via flow cytometry, and a T cell population with 95% purity was used for the following experiments. The study protocol was approved by the Institutional Review Board (IRB) of Chengdu Medical College.

### 3.4. Western Blot Analysis

The cells (5 × 10^6^) were lysed for 30 min in ice-cold lysis buffer (Bioteke Corp., Beijing, China). The cell lysates were cleared using centrifugation for 15 min at 15,000 rpm and used for immunoprecipitation. The proteins (50 μg/lane) were resolved using sodium dodecyl sulphate–polyacrylamide gel electrophoresis (SDS-PAGE), and the proteins were transferred to a PVDF membrane. The membranes were blocked with a 5% milk solution for 1 h and primary antibodies against the H+/K+-ATPases Beta-subunit (ATP4B) were used. The blots were developed using ECL chemiluminescence reagents (Millipore Corp., Bedford, MA, USA).

### 3.5. Measurement of ATPases Activity in T Cells

The cells (5 × 10^7^) were collected by centrifugation at 600× *g* for 5 min at 4 °C and washed with ice-cold 0.9% saline solution. Afterwards, the cells were homogenised in an ice-cold dounce tissue grinder, and the homogenate was transferred into tubes with final BMT-1 concentrations of 0, 25, 50, 100 μM, and DMSO was used as negative control. The samples were incubated for 10 min at 37 °C. To analyse the activity of the H+/K+-ATPase, the OD value at 660 nm was detected according to the procedure for the H+/K+-ATPase kit (Nanjing Jiancheng Biochemical Institute, Nanjing, China). The activity of H+/K+-ATPases (U/mg-prot) = (OD_test − OD_control)/OD_standard × Standard Concentration (0.5 μmol/mL) × (60 min/10 min) × dilution ratio (4.8)/protein concentration (mg/mL).

### 3.6. Evaluation of Cytosolic pH

The pH standard buffer solutions were prepared as previously described [24], and the pH of the solution was regulated to 5.8, 6.2, 6.6, 7.0, and 7.4. The first step was to establish a standard curve. The cells were cultured for 24 h in six-well plates at 5 × 10^5^ cells per well. Next, the upper medium was removed, and the cells were washed with PBS twice. Subsequently, the BCECF-AM (final concentration = 5 μM) was added, and the cells were incubated for 30 min. The supernatant was removed, and the cells were washed twice by the pH standard buffer solutions at different pH values. Afterward, nigericin was added to each well to a final concentration of 10 μM, and the cells were incubated for 15 min under normal conditions. The BCECF fluorescence intensity was recorded using flow cytometry. The effects of BMT-1 on the cytosolic pH were evaluated by flow cytometry using the pH-sensitive fluorescent probe BCECF-AM. Approximately 1 × 10^6^ cells were incubated at 37 °C for 30 min in 1 mL of RPMI containing 1 μmol/L BCECF-AM. The cells were washed in HBSS, placed on ice, and analysed by using the BD Accuri C6 Flow Cytometer (Accuri Cytometers, Ann Arbor, MI, USA) and CFlow software (version 1.0.227.4, BD Accuri) to collect the emission of BCECF-AM in the FL1channels. Finally, the relative cytosolic pH of individual cells was calculated according to the pHi standard.

### 3.7. Proliferation Testing

The purified human T cells were incubated with CFSE in PBS at a final concentration of 2.5 μM for 10 min. The CFSE-labelled cells were stimulated with 5 μg/mL PHA or plate-bound anti-CD3 (2 μg/mL) plus soluble anti-CD28 (1 μg/mL). The cells were cultured for 5 days in the presence or absence of varying concentrations of BMT-1. Rapamycin was used as a positive control. The cells were sorted based on the CFSE fluorescence. Flow cytometric analysis was performed using a FACScan flow cytometer (Becton Dickinson, San Jose, CA, USA) and analysed using CELLQuest software (Becton Dickinson). Methods using CFSE labelling were employed to calculate the absolute number of mitotic events occurring in the culture.

### 3.8. Viability of Lymphocytes and BMT-1 Exposure

To test the cytotoxicity of BMT-1, PBMCs were treated with different concentrations of BMT-1 for 72 h. The viable cell density was then measured using a BD Accuri C6 Flow Cytometer (BD Accuri). The data were analysed using CFlow software (version 1.0.227.4, BD Accuri). The results were expressed as the mean percentage survival ± SD for four replicates.

### 3.9. Cell Cycle Analysis

The density of purified human T cells was adjusted to 1 × 10^6^ cells/mL before use of the cells. The cell suspension (1 mL) was placed in a six-well flat-bottomed plate. The cells were stimulated with 5 μg/mL PHA or plate-bound anti-CD3 (2 μg/mL) plus soluble anti-CD28 (1 μg/mL). BMT-1 was added to the cells. The plates were incubated under a 5% CO2-air humidified atmosphere at 37 °C for 3 days. The cells were harvested using centrifugation, washed in PBS, and fixed in 70% ethanol for 30 min at −20 °C. After washing the cells once with PBS, DNA was stained with propidium iodide (20 μg/mL) containing 100 μg/mL of ribonuclease A for 30 min. Flow cytometric analysis was conducted with a BD Accuri C6 Flow Cytometer (BD Accuri), and data were analysed using CFlow software (version 1.0.227.4, BD Accuri).

### 3.10. The Effect of BMT-1 on the Expression of CD25 (IL-2 Receptor Chain) and IL-2 on Human T Cells Stimulated with Anti-CD3/CD28

The CD25 expression in lymphocytes was measured 48 h after the T cells were stimulated with anti-CD3/CD28 antibodies. The cell staining data were acquired using a BD Accuri C6 Flow Cytometer (BD Accuri) and analysed using CFlow software (version 1.0.227.4, BD Accuri). The IL-2 concentrations in the culture supernatants were determined using ELISA via the Quantikine immunoassays manufactured by eBioscience Systems (San Diego, CA, USA). Recombinant IL-2 was used as the standard. The assays were performed according to the protocol provided by eBioscience.

### 3.11. Inhibition of Human IL-2 Dependent PBMCs Blast Proliferation

The PBMCs were cultured at 2 × 10^6^/mL in media; proliferation was induced by adding 5 μg/mL PHA. After 3 days at 37 °C in 5% CO_2_, the cells were washed three times in media and resuspended to reach a density of 1 × 10^6^/mL in media containing 100 units/mL human recombinant IL-2 (eBioscience). After one week, the cells were IL-2-dependent. The cells can be maintained for up to 3 weeks by feeding with equal volumes of media plus 100 units/mL IL-2 twice a week [29]. To assess the ability of a compound to inhibit the proliferation of IL-2-dependent cells, the IL-2-dependent cells were washed three times. After incubation with CFSE in PBS at 2.5 μM for 10 min, the cells were plated (1 × 10^6^/mL per well/0.1 mL, with 100 units/mL IL-2) in a flat-bottom 96-well microtiter plate. Using a 10 mM stock of BMT-1 in dimethyl sulphoxide (DMSO), 2-fold serial dilutions of the compound were added in triplicate wells starting at 2.5 μM. The plates were incubated at 37 °C under 5% CO_2_ for 5 days. The inhibition of the IL-2-dependent cells was then measured based on the CFSE fluorescence.

## 4. Conclusions

In summary, BMT-1 inhibits the proliferation of anti-CD3/CD28-stimulated T cells by interfering with the H+/K+-ATPases and down-regulates the intracellular pHi. BMT-1 exhibits promising *in vitro* immunosuppressive activity. The opportunity exists to prepare a wide range of compounds by introducing various substituents to this structure. BMT-1 may be used as a lead compound for the design and development of new immunosuppressive agents. Therefore, further studies, including the design, synthesis, and analysis of subsequent derivatives as well as the extension of immunosuppressive activity should be performed.
